# Supplementary figures and images for: KIF2C regulates synaptic plasticity and cognition in mice through dynamic microtubule depolymerization
Source: eLife. 2022 Feb 9;11:e72483. doi: 10.7554/eLife.72483 (PMC8828051; doi:10.7554/eLife.72483)

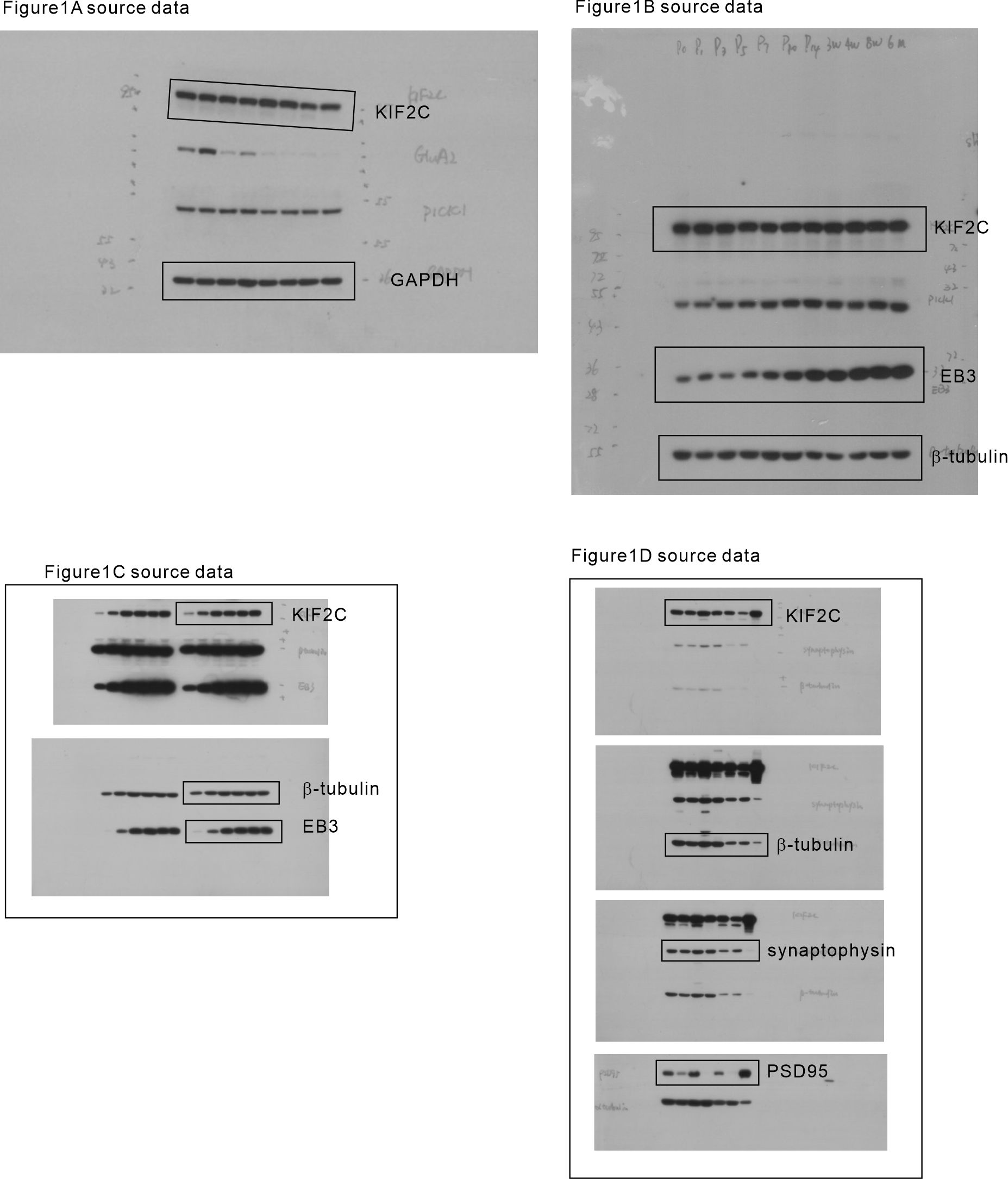

Supplement: Figure 1—source data 1. [file elife-72483-fig1-data1.zip › Figure 1-source data.jpg]

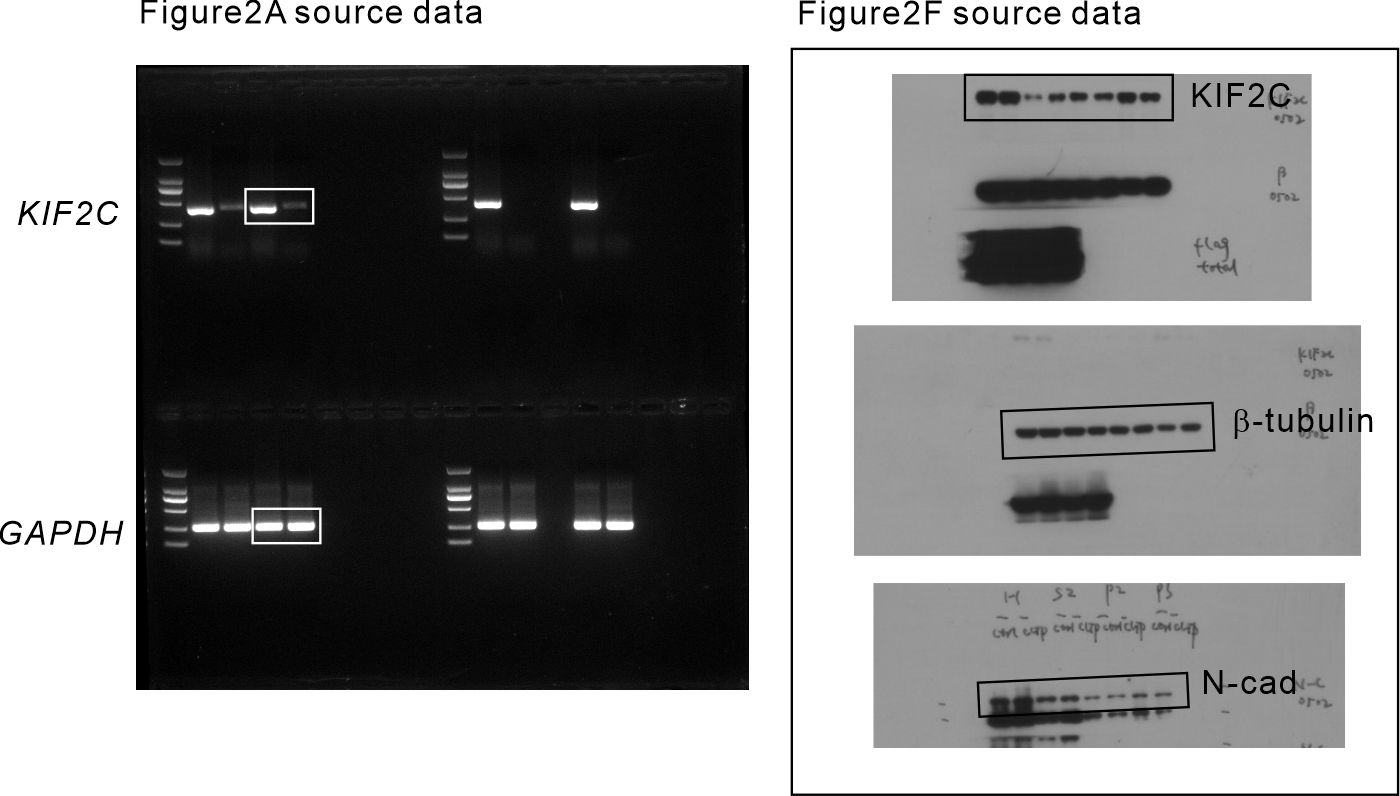

Supplement: Figure 2—source data 2. [file elife-72483-fig2-data2.zip › Figure 2-source data 2.jpg]

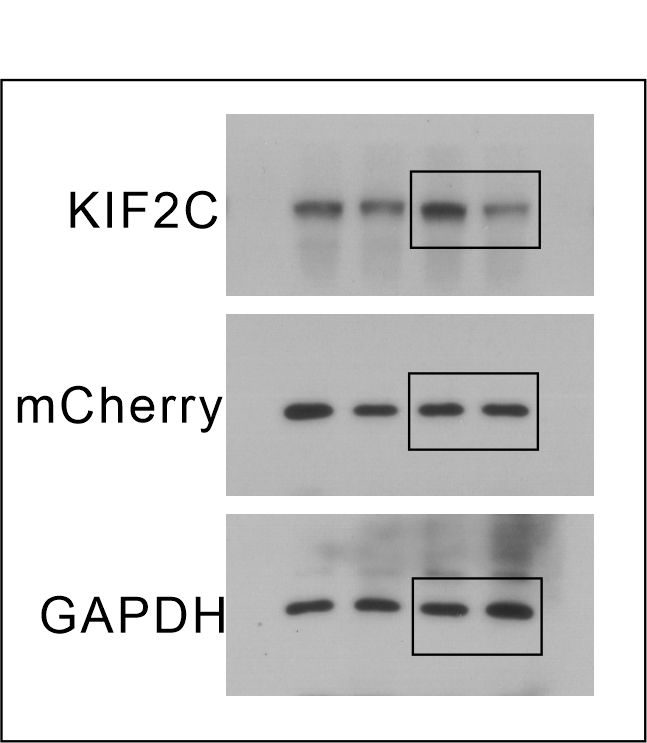

Supplement: Figure 2—figure supplement 1—source data 2. [file elife-72483-fig2-figsupp1-data2.zip › Figure2- figure supplement1- source data2.jpg]

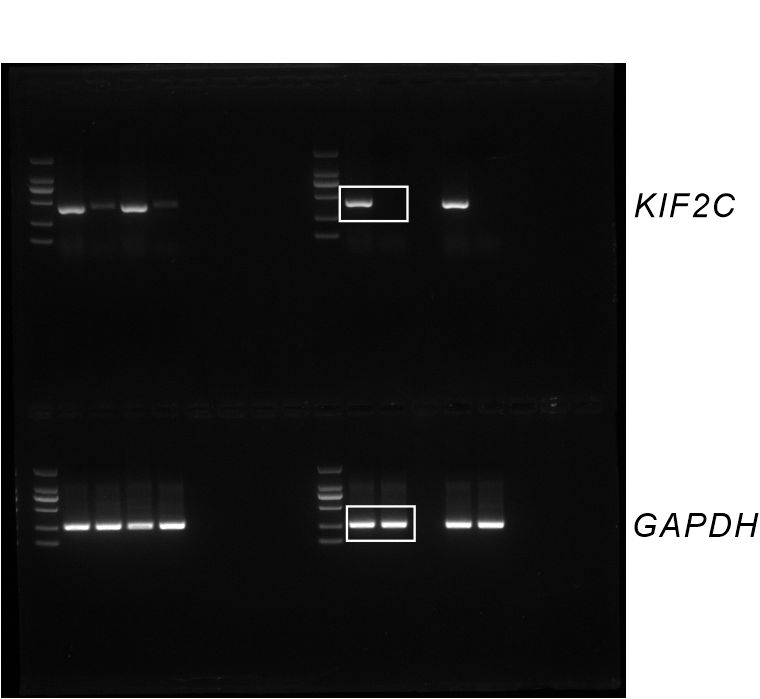

Supplement: Figure 4—figure supplement 1—source data 2. [file elife-72483-fig4-figsupp1-data2.zip › Figure4- figure supplement1- source data2.jpg]

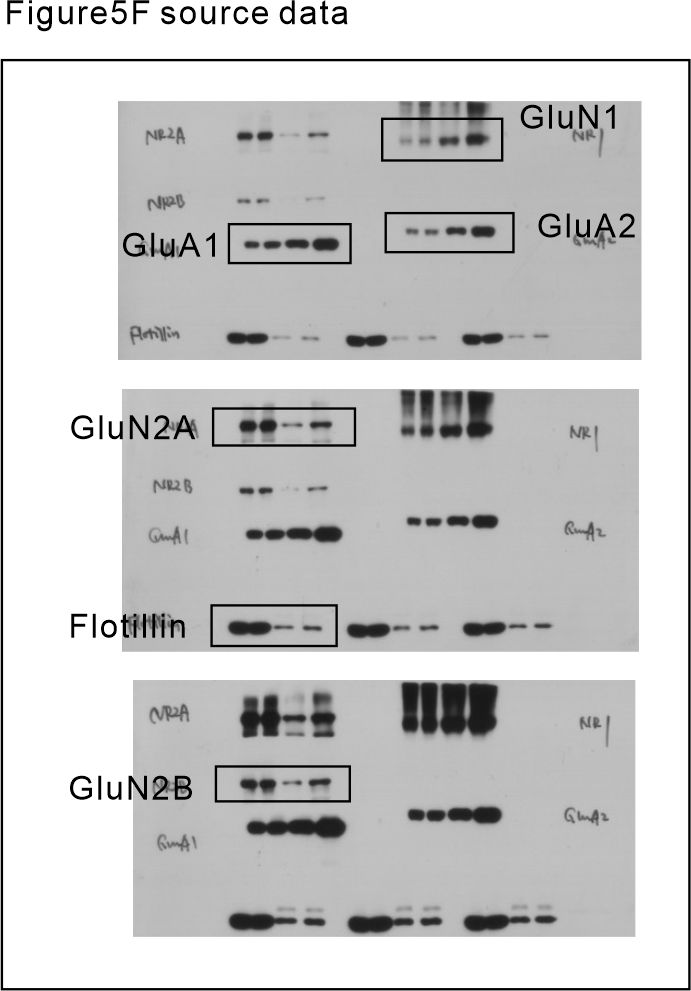

Supplement: Figure 5—source data 2. [file elife-72483-fig5-data2.zip › Figure 5- source data2.jpg]

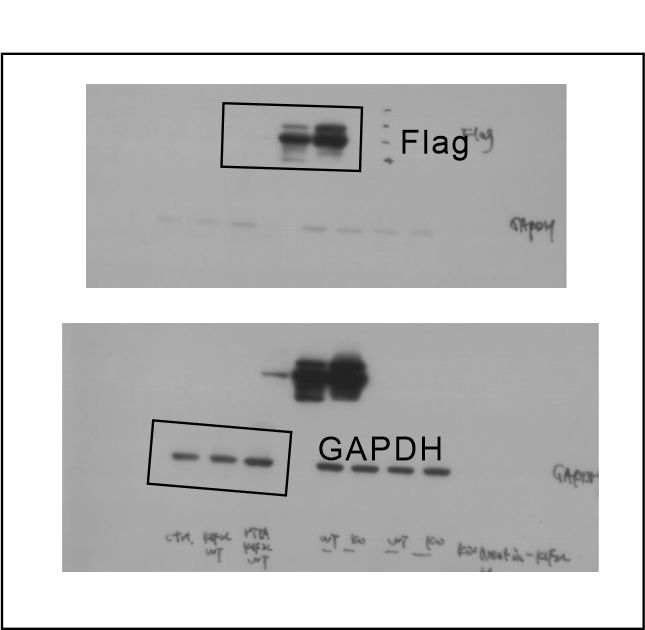

Supplement: Figure 6—figure supplement 1—source data 1. [file elife-72483-fig6-figsupp1-data1.zip › Figure6- figure supplement1- source data1.jpg]
